# Safety and Efficacy of Direct Oral Anticoagulants Apixaban and Rivaroxaban Versus Standard Therapy for VTE Prophylaxis Post Cancer Surgery—A Network Meta-Analysis of Randomized Clinical Trials

**DOI:** 10.3390/jcm14061811

**Published:** 2025-03-07

**Authors:** Alaa Shahbar, Abdulaziz Alawlqi, Abdullah Alhifany, Afnan Noor, Abdulaali R. Almutairi, Mohammed Alnuhait

**Affiliations:** 1 Pharmaceutical Practices Department, College of Pharmacy, Umm Al-Qura University, Makkah 21955, Saudi Arabia; 2 Taif Health Cluster, King Faisal Medical Complex, Clinical Pharmacy Services, Taif 26514, Saudi Arabia; 3 Pharmaceutical Care Department, King Faisal Specialist Hospital & Research Center, Jeddah 22234, Saudi Arabia; 4 Saudi Food and Drug Authority, Riyadh 7148-13513, Saudi Arabia

**Keywords:** anticoagulants, prophylaxis, post cancer surgery, VTE

## Abstract

**Background/Objectives**: Venous thromboembolism (VTE) is a major risk for cancer patients undergoing surgery due to hypercoagulability and surgical stress. Traditional low-molecular-weight heparins (LMWHs) are used as the standard of care for VTE prophylaxis, but subcutaneous administration often leads to suboptimal patient adherence. Direct oral anticoagulants (DOACs) are being explored as more convenient and effective alternatives. This study employed a network meta-analysis approach to comparatively assess the safety and efficacy of DOACS and LMWH in preventing VTE among cancer patients undergoing oncologic surgery. **Methods**: A systematic review and network meta-analysis were conducted. The search strategy included randomized controlled trials (RCTs) retrieved from databases such as CLINICALTRIAL.GOV, MEDLINE, and EMBASE. The search encompassed studies published up to October 2023 and compared the efficacy and safety of DOACs with LMWHs in patients undergoing cancer surgery. The primary outcome was the incidence of VTE, and the secondary outcomes included the incidences of major bleeding events (MB) and clinically relevant non-major bleeding (CRNMB). **Results**: A network meta-analysis of four randomized controlled trials (RCTs) involving 1600 cancer surgery patients was conducted. No statistically significant differences in VTE rates were observed between DOACs and LMWHs. While rivaroxaban 10 mg once daily for 30 days significantly reduced VTE risk compared to placebo (RR: 0.27, 95% CI: 0.08–0.95), no significant differences were found in major or clinically relevant non-major bleeding risks between DOACs and LMWH or placebo. **Conclusions**: This network meta-analysis provides evidence supporting the use of DOACs, specifically apixaban and rivaroxaban, as safe and efficacious alternatives to LMWHs for VTE prophylaxis in cancer patients undergoing surgery. The oral administration and reduced monitoring requirements associated with DOACs address the limitations inherent to LMWHs, potentially improving patient adherence. These findings emphasize the need for additional head-to-head trials and long-term studies further to solidify their role in this high-risk patient population.

## 1. Introduction

Venous thromboembolism (VTE) ranks among the top three causes of death worldwide. It is frequently associated with cancer patients, either due to the disease itself or because of chemotherapy and surgery (Lunenfeld et al., 2013 [1]). The development of VTE in cancer patients can be attributed to several factors, including immobilization, age, trauma, hormonal therapy, surgery, central venous catheters, chemotherapy, and the hypercoagulable state induced by tumor cells (Karimi et al., 2010 [2]).

Furthermore, ECOG scores and patient gender, particularly female gender, have been linked to an increased risk of thrombosis (X. Wang et al., 2019 [3]; Havas & Favaloro, 2017 [4]). Among cancer patients receiving chemotherapy, the annual VTE incidence is approximately 10.9%, underscoring its clinical relevance (Otten, 2004 [5]). Cancer and oncologic surgery activate the platelet coagulation cascade and promote the overproduction of procoagulant components, predisposing patients to VTE (Suzuki-Inoue, 2019 [6]).

For patients undergoing major abdominal or pelvic cancer surgery, the American Society of Clinical Oncology (ASCO) and the American College of Chest Physicians (ACCP) recommend extended pharmacological postsurgical thromboprophylaxis in high-risk patients without high risk of bleeding for four weeks following preoperative low-molecular-weight heparin (LMWH) therapy (Gould et al., 2012 [7]; Key et al., 2023 [8]). LMWHs are currently the standard of care for VTE prophylaxis in patients undergoing surgery for pelvic or abdominal malignancies (Key et al., 2023 [8]).

However, discomfort associated with subcutaneous injections and adverse effects, such as local pain and bruising, pose challenges to continued LMWH therapy (Li et al., 2021 [9]). As a result, nearly 32% of the patients do not adhere to long-term parenteral LMWH treatment (Gezelius et al., 2019 [10]). Direct oral anticoagulants (DOACs), such as apixaban and rivaroxaban, offer a potentially safe and effective alternative to LMWH for reducing the risk of thrombotic events (Schrag et al., 2023 [11]). These agents have recently been shown to be at least as effective and safe as LMWH in VTE recurrence and bleeding incidence in post-oncological procedures in cancer patients (Zhou et al., 2024 [12]).

This study uses a network meta-analysis approach to assess the safety and efficacy of apixaban and rivaroxaban compared with the standard of care (LMWH) for VTE prophylaxis in cancer patients undergoing surgery. This method allows for the simultaneous evaluation of DOACs (apixaban and rivaroxaban) versus LMWH by combining both direct and indirect comparisons across network studies. Given the limited research on the effectiveness and safety of DOACs for VTE prevention in cancer patients following oncologic surgery, we conducted a systematic review and network meta-analysis (NMA) of the available randomized clinical trial (RCT) data to better understand the effectiveness and safety of DOACs in this population and guide the selection of appropriate medications for VTE prevention post-surgery.

## 2. Method

### 2.1. Study Design

This study was conducted as a systematic review and network meta-analysis to compare the safety and efficacy of direct oral anticoagulants (DOACs)—apixaban, rivaroxaban, and edoxaban—against the standard thromboprophylaxis options, including low-molecular-weight heparin (LMWH), warfarin, and aspirin, in cancer patients undergoing surgery. Network meta-analysis was selected as the most appropriate methodology due to its ability to integrate direct and indirect evidence across multiple interventions.

### 2.2. Outcomes

1. Primary Outcome:

The primary outcome was the occurrence of venous thromboembolism (VTE), including deep vein thrombosis (DVT) and pulmonary embolism (PE).

2. Secondary Outcomes:

- Incidence of major bleeding events.
- Incidence of clinically relevant non-major bleeding (CRNMB) events.

### 2.3. Data Collection Methods

A comprehensive search of CLINICALTRIAL.GOV, MEDLINE, and EMBASE databases was performed to identify randomized controlled trials (RCTs) from inception through October 2023. The search strategy employed specific keywords and controlled vocabulary terms, including “apixaban”, “rivaroxaban”, “edoxaban”, “LMWH”, “warfarin”, and “aspirin”. Filters were applied to restrict the search to human studies and RCTs. In addition to database searches, reference lists of the relevant articles were reviewed to identify additional eligible studies. Titles and abstracts of all the retrieved articles were screened, and full-text reviews were conducted for potentially eligible studies. Studies not addressing the research question were excluded during the screening process.

### 2.4. Eligibility Criteria

1. Inclusion Criteria:

- RCTs evaluating primary thromboprophylaxis in adult cancer patients undergoing surgery.
- Studies involving anticoagulants (apixaban, rivaroxaban, edoxaban, LMWH, warfarin, or aspirin).
- A follow-up period exceeding 30 days.

2. Exclusion Criteria:

- Non-RCT study designs.
- Studies involving patients with confirmed VTE events before enrollment.
- Trials not meeting the inclusion criteria.

### 2.5. Study Selection

**Figure 1:**
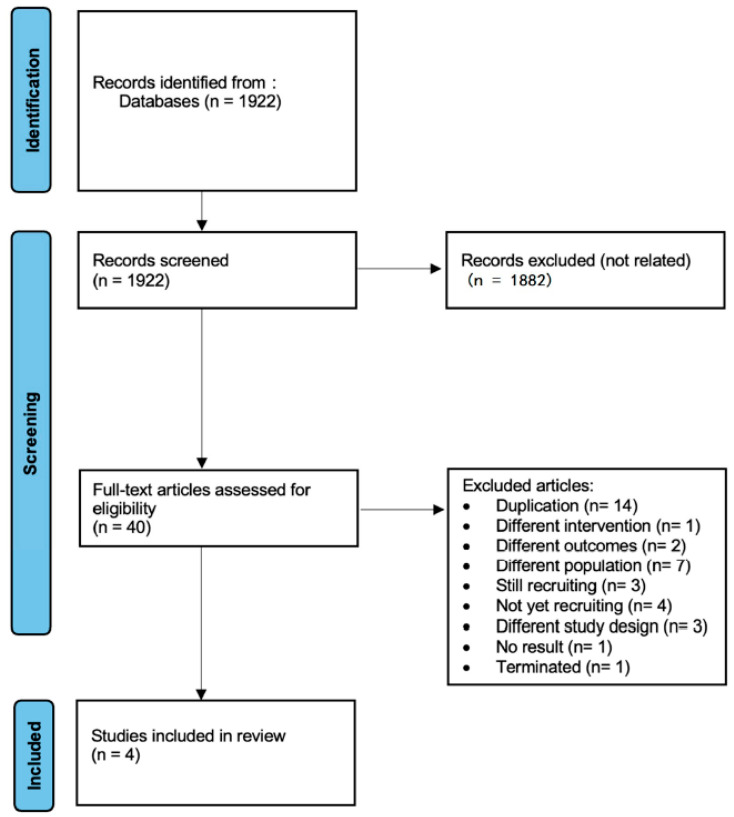
PRISMA flow diagram for identification of studies via databases.

Two independent reviewers assessed the eligibility of the studies by screening the titles, abstracts, and full texts. Disagreements were resolved by a third reviewer to ensure accuracy and consistency. A detailed depiction of the study selection process is provided in Figure 1.

### 2.6. Data Extraction and Quality Assessment

Data from the included studies were extracted using a standardized form that captured the following:

- Study characteristics (e.g., design, population, interventions, and comparators).
- Outcomes (e.g., VTE events, major bleeding, and CRNMB events).
- Follow-up duration.

Quality assessment of the included studies was performed using the Cochrane Risk of Bias tool, evaluating random sequence generation, allocation concealment, blinding, incomplete outcome data, and selective reporting.

### 2.7. Analysis Approach

The network meta-analysis was conducted to compare direct oral anticoagulants (DOACs) with standard thromboprophylaxis options, incorporating indirect comparisons where direct evidence was unavailable. Statistical heterogeneity was assessed using the I^2^ statistic, with thresholds of 25%, 50%, and 75% indicating low, moderate, and high heterogeneity, respectively. Sensitivity analyses were performed to ensure the robustness of the findings by systematically removing one study at a time while maintaining the same network diagram. The analysis was conducted using the MetaInsight shiny app, powered by the R netmeta package (V6.0.0), with applying the random-effect model network meta-analyses to account for potential heterogeneity (Owen et al., 2019 [13]). The analysis was based on the frequentist approach. Effect sizes were calculated for each outcome and expressed as risk ratios (RRs) with 95% confidence intervals (CIs).

## 3. Results

The initial literature search identified 1922 trials. After a thorough screening process, 1917 trials were excluded, leaving only 4 randomized controlled trials (RCTs) involving 1600 cancer patients who underwent surgical procedures to be included in our meta-analysis. The process of selecting and excluding articles for this network meta-analysis and systematic review is illustrated in the flowchart in Figure 1. Table 1 provides a detailed description of the included studies (Becattini et al., 2022 [14]; Guntupalli et al. [15], 2020; Longo de Oliveira et al., 2022 [16]; Zhao et al., 2023 [17]).

### 3.1. Efficacy Outcomes

#### VTE Occurrence

**Figure 2:**
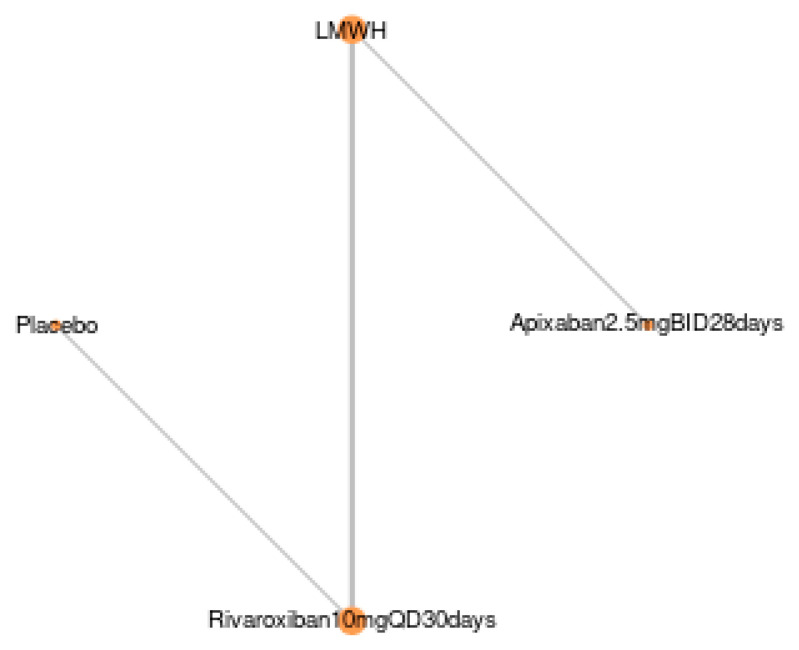
Network plot of included studies for the VTE recurrence outcome.

In the analyzed trials, a total of 89 out of 1600 patients experienced venous thromboembolism (VTE) events, with 34 events occurring among 805 patients treated with direct oral anticoagulants (DOACs), and 55 events among 795 patients receiving low-molecular-weight heparin (LMWH) or placebo. Figure 2 shows the comparisons involved in the network meta-analysis.

As presented in Table 2, rivaroxaban and apixaban demonstrated similar efficacy to LMWH in preventing VTE events in cancer patients following surgery. Notably, only rivaroxaban 10 mg once daily for 30 days showed a statistically significant reduction in VTE events compared to placebo (RR 0.27, 95% CI: 0.08–0.95). The network meta-analysis did not show a significant heterogeneity I^2^ = 0%. The sensitivity analyses by removing one study and keeping the same network diagram did not show a major change in the primary analysis (Supplemental Tables S1 and S2).

### 3.2. Safety Outcomes

#### 3.2.1. Major Bleeding

**Figure 3:**
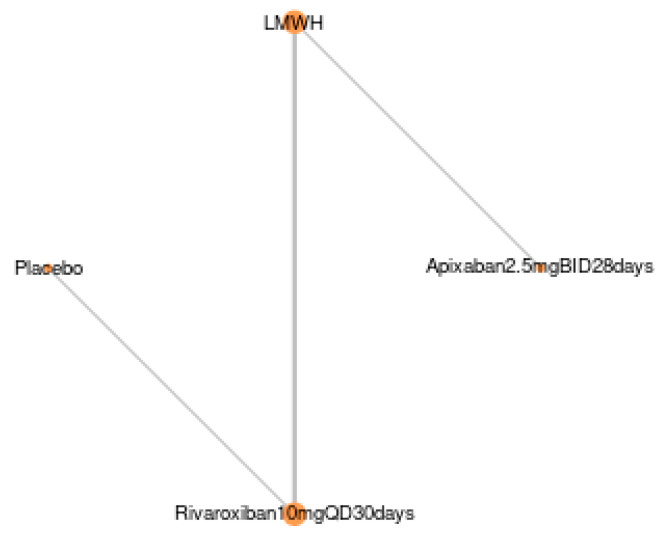
Network plot of included studies for the major bleeding outcome.

The analysis of clinical trial data revealed 36 major bleeding events among 1594 patients. Out of the 801 patients treated with DOAC, 22 had major bleeding compared to 14 events among 511 patients on LMWH. No major bleeding was observed among the placebo group. Four treatments were included in the network meta-analysis, as shown in Figure 3. As shown in Table 3, the pairwise comparisons between the four treatments did not show a statistical difference between the included therapies. Also, there was no statistically significant heterogeneity (I^2^ = 0%). Moreover, the sensitivity analyses were similar to the primary analysis (Supplemental Tables S4 and S5).

#### 3.2.2. Clinically Relevant Non-Major Bleeding

**Figure 4:**
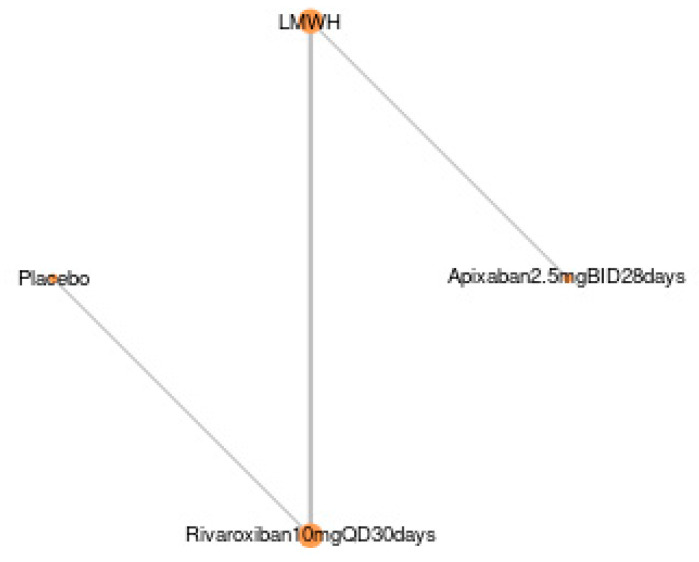
Network plot of included studies for CRNMB outcome.

A total of 48 clinically relevant non-major bleeding (CRNMB) events were recorded, with 20 associated with DOAC, 23 with LMWH, and 5 with placebo. There were four treatments included in the network meta-analysis (Figure 4). As shown in Table 4, all the pairwise comparisons did not show any statistically significant differences in reducing the risk of CNRMB. Although heterogeneity was high with I^2^ = 73% in the primary analysis, the sensitivity analyses by removing the Longo de Oliveira et al. [16] study, which has three NCRMB events in the LMWH arm compared to no event in the rivaroxiban arm, yielded I^2^ = 0% and a significant reduction in NCRMB risk in apixaban patients compared to placebo (RR 0.11, 95%CI 0.00, 0.97) (Supplemental Table S5). The sensitivity analysis which involved removing the Zhao et al. study [17], which has five NCRMB events in the rivaroxiban arm compared to one event in the LMWH arm, reduced heterogeneity to I^2^ = 0% with no statistical difference between the therapies in reducing the risk of NCRMB (Supplemental Table S6).

### 3.3. Study Quality

**Figure 5:**
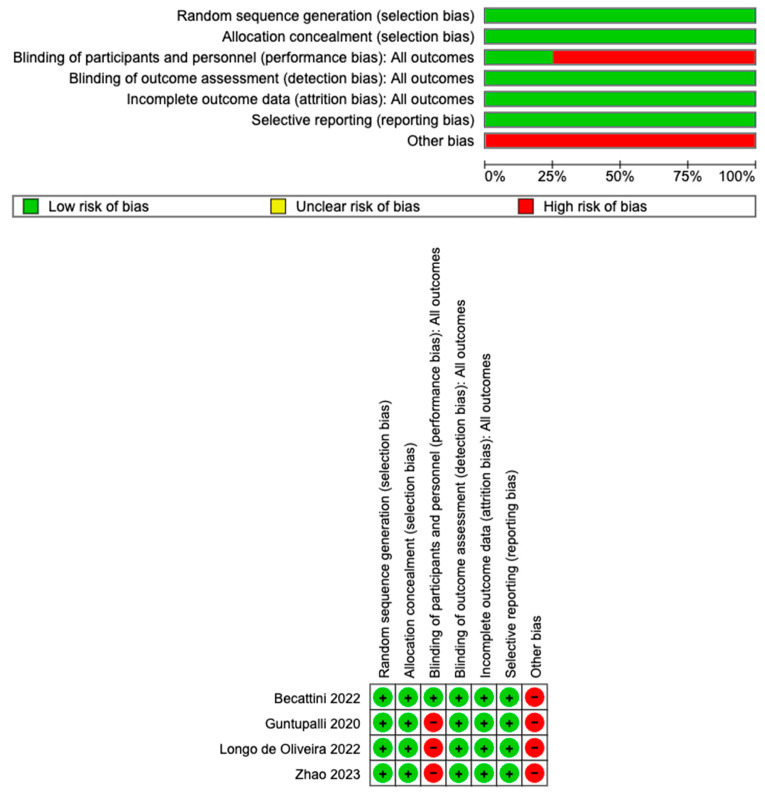
Risk of bias assessment [14,15,16,17].

All of the included studies provided details on the randomization techniques and allocation concealment. While several studies employed an open-label design, Becattini et al. (2022) [14] stood out as a double-blinded, randomized controlled trial. Outcome assessments across the studies were conducted by independent, centralized committees blinded to the treatment groups, mitigating detection bias. All the studies adhered to the planned analysis approaches—either intention-to-treat or per-protocol—thereby reducing the impact of patient attrition and incomplete outcome data. Figure 5 However, other biases were noted across the studies. Guntupalli et al. (2020) [15] and Longo de Oliveira et al. (2022) [16] prematurely halted their studies due to lower-than-expected rates of venous thromboembolism (VTE), Becattini et al. (2022) [14] terminated early because the investigational drugs expired, while Zhao et al. (2023) [17] excluded patients who required extended anticoagulation therapy.

## 4. Discussion

This network meta-analysis and systematic review evaluated four randomized controlled trials focusing on the primary prevention of venous thromboembolism (VTE) in cancer patients following oncological surgery. The analysis compared the safety and efficacy of direct oral anticoagulants (DOACs), apixaban and rivaroxaban, to low-molecular-weight heparin (LMWH) across key outcomes: VTE prevention, major bleeding, and clinically relevant non-major bleeding (CRNMB). Our findings indicate that the risk of developing VTE with rivaroxaban and apixaban was comparable to LMWH, demonstrating non-inferiority to LMWH.

Importantly, neither DOAC significantly increased the incidence of major bleeding or CRNMB events, underscoring their safety profile in this high-risk population. These results align with the current guidelines from the American Society of Clinical Oncology (ASCO) and the National Comprehensive Cancer Network (NCCN), which recommend LMWH as the first-line agent for post-operative VTE prophylaxis while acknowledging DOACs as viable alternatives under specific circumstances (Key et al., 2023 [8]; NCCN, 2024 [18]).

The practical advantages of DOACs, including oral administration and reduced monitoring requirements, position them as more patient-friendly options compared to LMWH. Poor adherence to LMWH due to discomfort from subcutaneous injections and frequent monitoring is well documented, with nearly 32% of the patients discontinuing therapy prematurely (Li et al., 2021 [9]; Gezelius et al., 2019 [10]). Thus, rivaroxaban and apixaban could address these barriers, potentially improving compliance with thromboprophylaxis regimens in cancer patients. The high heterogeneity observed in the CRNMB analysis was driven by the differences in the risk of CRNMB among rivaroxaban and LMWH in two studies where one yielded a higher rate in LMWH while the other one reported a higher rate of CRNMB among rivaroxaban. Both studies did not show a statistically significant difference between rivaroxaban and LMWH in the risk of CRNMB. Despite these promising findings, certain limitations must be acknowledged. First, due to a lack of clinical trials focusing on postoperative cancer patients, this analysis did not include other FDA-approved DOACs, such as edoxaban and dabigatran. Second, the number of included trials was limited, reflecting the relative scarcity of research in this specific population.

Furthermore, potential biases in the included studies may have influenced the results. For instance, some studies experienced early termination due to logistical challenges or regulatory changes. Guntupalli et al. (2020) [15] and Longo de Oliveira et al. (2022) [16] ended prematurely because of lower-than-expected VTE rates, while Zhao et al. (2023) [17] excluded patients requiring extended anticoagulation therapy. These factors highlight the need for more robust, high-quality trials. Future research should prioritize head-to-head comparisons of all DOACs and LMWH in this population to provide a more comprehensive understanding of their relative efficacy and safety. Additionally, long-term studies assessing adherence, cost-effectiveness, and patient-reported outcomes are warranted to support guideline development and clinical decision making.

## 5. Conclusions

This study adds to the growing evidence supporting the use of DOACs for VTE in cancer patients following surgery. Both rivaroxaban and apixaban were shown to be as effective and safe as low-molecular-weight heparin, with the added advantage of easier administration, which may improve patient adherence. These findings provide practical insights to help clinicians make informed decisions and enhance VTE prevention strategies in this vulnerable patient group.

## Figures and Tables

**Table 1:** Characteristics of included randomized clinical trials in meta-analysis.

Study	(Guntupalli et al., 2020) [15]	(Longo de Oliveira et al., 2022) [16]	(Zhao et al., 2023) [17]	Becattini et al., 2022 [14]
Year	2019	2022	2023	2022
Follow-up period	90 days	30 days	30 days	90 days
Study design	Prospective, randomized, open-blinded clinical trial	Open-label, randomization, active-controlled trial	Single-blind, noninferiority, randomized clinical trial	Double-blind, randomized clinical trial
N	400	228	403	569
Type of procedure	Laparotomy and laparoscopy	Major gynecological cancer surgery	Anatomical lobectomy or segmentectomy	Laparoscopic surgery
Intervention	Apixaban 2.5 twice daily (*n* = 204) for 28 days	Rivaroxaban 10 mg once daily (*n* = 114) for 30 days	Rivaroxaban 10 mg once daily not more than 7 days (*n* = 200)	Rivaroxaban 10 mg once daily for 30 days (*n* = 287)
Control	Enoxaparin 40 mg SC (*n* = 196) for 28 days	Enoxaparin 40 mg SC once daily (*n* = 114) for 30 days	Nadroparin 38 unit/kg for 3 days then 57 units/kg until discharge (*n* = 203)	Placebo (*n* = 282)
Type of cancer	Gynecologic cancer	Gynecologic cancer	Lung Cancer	Colorectal
VTE in intervention arm	2 (1.0%)	4 (3.51%)	25 (12.5%)	3 (1.0%)
VTE in control arm	3 (1.5%)	5 (4.39%)	36 (17.7%)	11 (3.9%)
Major bleeding in intervention arm	1 (0.5%)	0	19 (9.7%)	2 (0.7%)
Major bleeding in control arm	1 (0.5%)	0	13 (6.6%)	0
CRNMB in intervention arm	12 (5.88%)	0	5 (2.6%)	3 (1.0%)
CRNMB in control arm	19 (9.69%)	3 (2.63%)	1 (0.5%)	5 (1.8%)
Early termination	Prematurely halted their study due to lower-than-expected rates of VTE	Prematurely halted their study due to lower-than-expected rates of VTE	No early termination	Prematurely halted because the investigational drugs expired

**Table 2:** Comparative efficacy of direct oral anticoagulants, LMWH, and placebo for VTE occurrence.

Rivaroxiban10 mg QD 30 Days			
1.12 [0.18, 6.98]	Apixaban		
0.72 [0.46, 1.11]	0.64 [0.11, 3.79]	LMWH	
0.27 [0.08, 0.95]	0.24 [0.03, 2.23]	0.37 [0.10, 1.43]	Placebo

Treatments are ranked from best to worst along the leading diagonal. Below the leading diagonal are estimates from network meta-analyses (column versus row).

**Table 3:** Comparison of major bleeding among direct oral anticoagulants, LMWH, and placebo.

Placebo			
0.31 [0.01, 6.82]	LMWH		
0.32 [0.00, 20.32]	1.04 [0.07, 16.52]	Apixaban 2.5 mg BID for 30 days	
0.20 [0.01, 4.22]	0.67 [0.34, 1.31]	0.64 [0.04, 11.04]	Rivaroxaban 10 mg QD for 30 days

**Table 4:** Comparison of clinically relevant non-major bleeding (CRNMB) among direct oral anticoagulants, LMWH, and placebo.

Apixaban 2.5 mg BID for 30 days			
0.61 [0.30, 1.22]	LMWH		
0.41 [0.06, 2.62]	0.67 [0.12, 3.78]	Rivaroxaban 10 mg QD for 30 days	
0.24 [0.02, 2.50]	0.39 [0.04, 3.70]	0.59 [0.14, 2.44]	Placebo

## Data Availability

The authors confirm that the data supporting the findings of this study are available within the article.

## Supplementary Materials

The following supporting information can be downloaded at: https://www.mdpi.com/article/10.3390/jcm14061811/s1, Table S1: Sensitivity analysis for the occurrence of venous thromboembolism (VTE) By removing (Zhao et al. study [17]); Table S2: Sensitivity analysis for the occurrence of venous thromboembolism (VTE) By removing (Longo de Oliveira et al., [16] study); Table S3: Sensitivity analysis for the occurrence of major bleeding (MB) By removing (Zhao et al. study [17]); Table S4: Sensitivity analysis for the occurrence of major bleeding (MB) By removing (Longo de Oliveira et al., study [16]); Table S5: Sensitivity analysis for the occurrence of clinical related non-major bleeding (CRNMB) By removing (Longo de Oliveira et al., study [16]); Table S6: Sensitivity analysis for the occurrence of clinical related non-major bleeding (CRNMB) By removing (Zhao et al. study [17]).

## References

[1] Lunenfeld B., Stratton P. (2013). The clinical consequences of an ageing world and preventive strategies. Best Pract. Res. Clin. Obstet. Gynaecol..

[2] Karimi M., Cohan N. (2010). Cancer-associated thrombosis. Open Cardiovasc. Med. J..

[3] Wang X., Wang S., Morse M.A., Jiang N., Zhao Y., Song Y., Zhou L., Huang H., Zhou X., Hobeika A. (2019). Prospective randomized comparative study on rivaroxaban and LMWH for prophylaxis of post-apheresis thrombosis in adoptive T cell immunotherapy cancer patients. J. Thromb. Thrombolysis.

[4] Hvas A.M., Favaloro E.J. (2017). Gender related issues in thrombosis and hemostasis. Expert Rev. Hematol..

[5] Otten H.M., Mathijssen J., ten Cate H., Soesan M., Inghels M., Richel D.J., Prins M.H. (2004). Symptomatic venous thromboembolism in cancer patients treated with chemotherapy: An underestimated phenomenon. Arch. Intern. Med..

[6] Suzuki-Inoue K. (2019). Platelets and cancer-associated thrombosis: Focusing on the platelet activation receptor CLEC-2 and podoplanin. Blood.

[7] Gould M.K., Garcia D.A., Wren S.M., Karanicolas P.J., Arcelus J.I., Heit J.A., Samama C.M. (2012). Prevention of VTE in nonorthopedic surgical patients: Antithrombotic therapy and prevention of thrombosis: American College of Chest Physicians Evidence-Based Clinical Practice Guidelines. Chest.

[8] Key N.S., Khorana A.A., Kuderer N.M., Bohlke K., Lee A.Y.Y., Arcelus J.I., Wong S.L., Balaban E.P., Flowers C.R., Gates L.E. (2023). Venous Thromboembolism Prophylaxis and Treatment in Patients With Cancer: ASCO Guideline Update. J. Clin. Oncol..

[9] Li Y., Dong S., Wang P., Sun J., Jiang H., Liu F. (2021). Influence of low-molecular-weight heparin injection sites on local bruising and pain: A systematic review and meta-analysis. J. Clin. Pharm. Ther..

[10] Gezelius E., Bendahl P.O., Gonçalves de Oliveira K., Ek L., Bergman B., Sundberg J., Strandberg K., Krämer R., Belting M. (2019). Low-molecular-weight heparin adherence and effects on survival within a randomised phase III lung cancer trial (RASTEN). Eur. J. Cancer.

[11] Schrag D., Uno H., Rosovsky R., Rutherford C., Sanfilippo K., Villano J.L., Drescher M., Jayaram N., Holmes C., Feldman L. (2023). Direct Oral Anticoagulants vs. Low-Molecular-Weight Heparin and Recurrent VTE in Patients With Cancer: A Randomized Clinical Trial. JAMA.

[12] Zhou H., Chen T.T., Ye L.L., Ma J.J., Zhang J.H. (2024). Efficacy and safety of direct oral anticoagulants versus low-molecular-weight heparin for thromboprophylaxis after cancer surgery: A systematic review and meta-analysis. World J. Surg. Oncol..

[13] Owen R.K., Bradbury N., Xin Y., Cooper N., Sutton A. (2019). MetaInsight: An interactive web-based tool for analyzing, interrogating, and visualizing network meta-analyses using R-shiny and netmeta. Res. Synth. Methods.

[14] Becattini C., Pace U., Pirozzi F., Donini A., Avruscio G., Rondelli F., Boncompagni M., Chiari D., De Prizio M., Visonà A. (2022). Rivaroxaban vs. placebo for extended antithrombotic prophylaxis after laparoscopic surgery for colorectal cancer. Blood.

[15] Guntupalli S.R., Brennecke A., Behbakht K., Tayebnejad A., Breed C.A., Babayan L.M., Cheng G., Ramzan A.A., Wheeler L.J., Corr B.R. (2020). Safety and Efficacy of Apixaban vs. Enoxaparin for Preventing Postoperative Venous Thromboembolism in Women Undergoing Surgery for Gynecologic Malignant Neoplasm: A Randomized Clinical Trial. JAMA Netw. Open.

[16] Longo de Oliveira A.L.M., de Oliveira Pereira R.F., Agati L.B., Ribeiro C.M., Kawamura Suguiura G.Y., Cioni C.H., Bermudez M., Pirani M.B., Caffaro R.A., Castelli V. (2022). Rivaroxaban Versus Enoxaparin for Thromboprophylaxis After major Gynecological Cancer Surgery: The VALERIA Trial: Venous thromboembolism prophylAxis after gynecoLogical pElvic cancer surgery with RIvaroxaban versus enoxAparin (VALERIA trial). Clin. Appl. Thromb./Hemost..

[17] Zhao M., Bao Y., Jiang C., Chen L., Xu L., Liu X., Li J., Yang Y., Jiang G., Li J. (2023). Rivaroxaban versus nadroparin for thromboprophylaxis following thoracic surgery for lung cancer: A randomized, noninferiority trial. Am. J. Hematol..

[18] National Comprehensive Cancer Network (2024). NCCN Guidelines Version 2.2024: Cancer-Associated Venous Thromboembolic Disease. https://www.nccn.org/guidelines/guidelines-detail?category=3&id=1423.

