# Supplementary material for: Safety and Efficacy of Direct Oral Anticoagulants Apixaban and Rivaroxaban Versus Standard Therapy for VTE Prophylaxis Post Cancer Surgery—A Network Meta-Analysis of Randomized Clinical Trials
Source: J Clin Med. 2025 Mar 7;14(6):1811. doi: 10.3390/jcm14061811 (PMC11943130; doi:10.3390/jcm14061811)
Supplement: Supplementary file 1 [file jcm-14-01811-s001.zip › jcm-3406025-supplementary.pdf]

Sensitivity analyses:

Table S1: Sensitivity analysis for the occurrence of venous thromboembolism (VTE) By removing (Zhao et al study)

Rivaroxiban10mgQD30days			
1.25 [0.14; 11.11]0.14	Apixaban		
0.80 [0.22; 2.90]	0.64 [0.11; 3.79]	LMWH	
0.27 [0.08; 0.95]	0.21 [0.02; 2.71]	0.33 [0.06; 2.04]	Placebo

Treatments are ranked from best to worst along the leading diagonal. Below the leading diagonal are estimates from network meta-analyses (column versus row)

Table S2: Sensitivity analysis for the occurrence of venous thromboembolism (VTE) By removing (Longo de Oliveira et al., study

Rivaroxiban10mgQD30days			
1.10 [0.17; 6.93]	Apixaban		
0.70 [0.44; 1.13]	0.64 [0.11; 3.79]	LMWH	
0.27 [0.08; 0.95]	0.24 [0.03; 2.27]	0.38 [0.10; 1.47]	Placebo

Treatments are ranked from best to worst along the leading diagonal. Below the leading diagonal are estimates from network meta-analyses (column versus row)

Table S3: Sensitivity analysis for the occurrence of major bleeding (MB) By removing (Zhao et al study)

Placebo			
0.31 [0.01; 6.82]	LMWH		
0.32 [0.00; 20.32]	1.04 [0.07; 16.52]	Apixaban2.5mgBID28days	
0.20 [0.01; 4.22]	0.67 [0.34; 1.31]	0.64 [0.04; 11.04]	Rivaroxiban10mgQD30days

Treatments are ranked from best to worst along the leading diagonal. Below the leading diagonal are estimates from network meta-analyses (column versus row)

Table S4: Sensitivity analysis for the occurrence of major bleeding (MB) By removing (Longo de Oliveira et al., study)

Placebo			
0.31 [0.01; 6.82]	LMWH		
0.32 [0.00; 20.32]	1.04 [0.07; 16.52]	Apixaban2.5mgBID28days	
0.20 [0.01; 4.22]	0.67 [0.34; 1.31]	0.64 [0.04; 11.04]	Rivaroxiban10mgQD30days

Treatments are ranked from best to worst along the leading diagonal. Below the leading diagonal are estimates from network meta-analyses (column versus row)

Supplementary Table S5: Sensitivity analysis for the occurrence of clinical related non-major bleeding (CRNMB) By removing (Longo de Oliveira et al., study)

Apixaban2_5mgBID28days			
0.58 [0.27; 1.23]	LMWH		
0.11 [0.01; 1.09]	0.19 [0.02; 1.65]	Rivaroxiban10mgQD30days	
0.07 [0.00; 0.97]	0.11 [0.01; 1.49]	0.59 [0.14; 2.47]	Placebo

Treatments are ranked from best to worst along the leading diagonal. Below the leading diagonal are estimates from network meta-analyses (column versus row)

Supplementary Table S6: Sensitivity analysis for the occurrence of clinical related non-major bleeding (CRNMB) By removing (Zhao et al study)

Rivaroxiban10mgQD30days			
0.59 [0.14; 2.44]	Placebo		
0.24 [0.01; 4.89]	0.40 [0.01; 11.38]	Apixaban2_5mgBID28days	
0.14 [0.01; 2.73]	0.24 [0.01; 6.42]	0.61 [0.30; 1.22]	LMWH
